# International Web-based consultation on priorities for translational breast cancer research

**DOI:** 10.1186/bcr1798

**Published:** 2007-11-22

**Authors:** Mitch Dowsett, Aron Goldhirsch, Daniel F Hayes, Hans-Joerg Senn, William Wood, Giuseppe Viale

**Affiliations:** 1 Academic Department of Biochemistry, Royal Marsden Hospital, London SW3 6JJ, UK.; 2 Department of Medical Oncology, Division of Medical Oncology, Dept. of Medicine, European Institute of Oncology, Via Ripamonti 435, Milan 20141, Italy.; 3 Department of Medicine, University of Michigan, 6312 Cancer Centre, 1500E Medical Centre Drive, Ann Arbor, MI, 48109-0942, USA.; 4 Centre for Tumour Detection, Treatment and Prevention (ZeTuP), Rorschacherstrsse 150, CH-9006, St Gallen, Switzerland.; 5 Department of Surgery, Emory University Hospital, 1364 Clifton Road NE B206, Atlanta, GA, 30322-1059, USA.; 6 Department of Pathology, European Institute of Oncology, Via Ripamonti 435, Milan 20141 Italy.

## Abstract

**Background:**

Large numbers of translational breast cancer research topics have been completed or are underway, but they differ widely in their immediate and/or future importance to clinical management. We therefore conducted an international Web-based consultation of breast cancer professionals to identify the topics most widely considered to be of highest priority.

**Methods:**

Potential participants were contacted via two large e-mail databases and asked to register, at a Web site, the issues that they felt to be of highest priority. Four hundred nine questions were reduced by a steering committee to 70 unique issues, and registrants were asked to select the 6 questions they considered to be the most important.

**Results:**

Votes were recorded from 420 voters (2,520 votes) from 48 countries, with 48% of voters coming from North America. Half of the voters identified themselves as clinicians, with the remainder being academics, research scientists, or pathologists. The highest priority was to identify molecular signatures to select patients who could be spared chemotherapy, which gained about 50% more votes than the second topic and was consistently voted top by voters in North America, Europe, and the rest of the world. Research scientists voted the determination of the role of stem cells in breast cancer development, progression, and treatment sensitivity as the most important issue, but this was considered the sixth priority for clinicians and fourth overall.

**Conclusion:**

This exercise may bring a greater focus of research resources onto issues voted as top priorities.

## Introduction

In recent years, there has been a major increase in the application of new scientific tools and knowledge to tissues and fluid samples from patients with cancer in an attempt to better understand the biology of the disease and the consequences that this biology may have for prognosis and response to treatment. The potential importance of this for increasing the individualisation of treatment is now sufficiently well recognised that few clinical trials are initiated without collection of tissues/fluids for future analysis, irrespective of the development of a hypothesis at the time. Of the solid tumours, breast cancer has received the greatest attention in this pursuit of translational research, and a number of resultant highly cited articles have affected our view of the disease [1,2].

It is clear, however, that although the new technologies such as expression array profiling are powerful tools, their untargeted application is likely to be a missed opportunity for delivering important clinical advances. Translational research frequently reflects the immediate scientific interests of the investigators and the specimens available to them as opposed to a specific attempt to address a question that has been identified as having the potential to advance the management of patients. The identification of high-priority questions for the breast cancer research community may be better obtained by wide consultation within that community than by local opinion based on the challenges posed and opportunities available to individual investigator groups. Such identification might result in a prioritisation of the respective issue in terms of research activity, funding priority, peer-reviewed publications, and symposia presentations.

The project reported here involved an international Web-based consultation of breast cancer practitioners and researchers in which opinion was sought to identify an initial set of topics/questions perceived to be of high importance. Voting was then conducted to determine a consensus position on the importance of the questions. The project was named the Top Ten program and arbitrarily gave particular emphasis to those 10 topics/questions receiving the most votes.

## Materials and methods

A steering committee composed of the authors of this paper was created to form a key advisory group on the format and execution of the consultation. An interactive Web site was developed [3] to allow participants to register and participate in the consultation. The program had the following key stages.

a. A primary target database of more than 4,000 potential participants was developed by consolidation of the contact e-mail addresses of registrants at the 2005 San Antonio Breast Cancer Symposium and the 2005 St Gallen Consensus Meeting on Primary Therapy of Early Breast Cancer. A letter describing the activity as 'a global program to identify the most important questions for the breast cancer community in the area of translational research' was e-mailed to members on the database in October 2006, inviting them (and any of their colleagues) to register on the Web site and contribute their own priorities for research with a view to voting later on a consolidated list. They were informed that the two-stage process was expected to take them no more than 20 minutes. It was declared that the list of the top ten priorities would be announced at the St Gallen Consensus Meeting in March 2007 and that a simultaneous announcement on the Web site would occur. All registrants would later receive a fuller report on the activity and its results. Registration required a contact e-mail address; identification as a clinician, pathologist, research scientist, or academic; and current home country. The list of registered priorities was cumulative and could be viewed by later registrants who were asked to examine the earlier list and place only previously unlisted issues. Logging of topics/questions ceased on 3 November 2006.

b. The steering committee reviewed the listings and created a consolidated list in two phases: (i) exclusion of issues that were not translational in their orientation or that were a clear duplication of another issue and (ii) reduction of markedly overlapping priorities to single unique topics.

c. On 8 January 2007, registrants were contacted again by e-mail to invite them to revisit the Web site and place their votes on the consolidated list of priorities on the Web site. Six votes in order of priority were required. Voting ceased on 9 February 2007.

d. Votes were counted and allocated scores as follows: top priority, 6 points; second priority, 5 points; third priority, 4 points; fourth priority, 3 points; fifth priority, 2 points; and sixth priority, 1 point. The scores were summed for each of the topics to create the consensus scoring. Descriptive analyses were performed to determine the relationship between profession and region with voting pattern.

e. The top ten priorities were announced at the St Gallen Consensus Meeting on 17 March 2007 and simultaneously on the Web site.

## Results

Four hundred and nine topics were logged by registrants but many were overlapping, and it was clear to the steering committee that the request to registrants to record only those topics that had not been previously logged was not fully adhered to. The initial consolidation to exclude topics that were clearly duplicated or that were not of a translational research nature reduced the number to 139. The aim and expectation of the steering committee had been to consolidate this further to no more than 30 unique topics. However, it became clear that it was not possible to reduce the number below 70 without complete exclusion of certain topics, which would have been contrary to one of the main goals of the project, namely to draw on widespread opinion unaffected by steering committee views. The full set of 70 questions is listed in Supplementary Table S1 (Additional File 1), which includes the categorisation (for example, chemotherapy, radiotherapy, and prognosis) that was designated to them.

Four hundred twenty registrants voted on the 70 topics. One hundred seventy-eight were from the US and a further 22 were from Canada, making a total of 48% of respondents being from North America. One hundred thirty-three (32%) were from Europe and the remaining 84 (20%) were from the rest of the world (RoW), with Japan making up the largest group. Registrants from a total of 48 countries voted. Two hundred nine (50%) of the registrants classed themselves primarily as clinicians, 101 (24%) as academics, 86 (20%) as research scientists, 14 (3%) as pathologists, and 10 registrants did not fit any of these categories. Whereas the proportions of research scientists and pathologists were relatively similar between North America and Europe, the proportions of academics and clinicians were not: in North America the proportions were 39% and 36%, respectively, and in Europe they were 59% and 71%, respectively.

**Figure 1:**
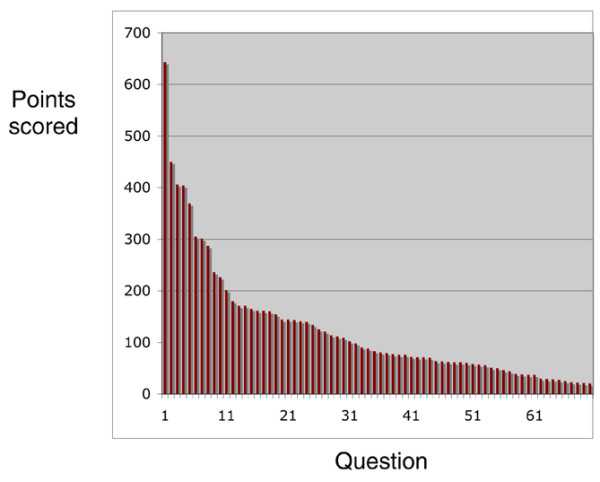
Distribution of votes overall among the 70 questions, showing the clear distinction of the highest priority. The identities and point allotments of the questions are presented in Supplementary Table S1 (Additional Data File 1).

The emphasis in this consultation was on the top ten priorities, but the focus on 10 was arbitrary, so the list of the top 15 is given in Table 1, together with the number of points scored. The weighting of each person's votes with 6 points downward to 1 point was also arbitrary. To ensure that this did not cause biases in the overall ranking, we also ranked the topics according to the number of votes cast irrespective of the order they were ranked in by individual voters and also according to the number of times the topic appeared as the top of individual rankings. These two alternate approaches had virtually no effect on the outcome and therefore are not presented. A histogram showing the overall voting pattern for the 70 questions based on the weighted points system is shown in Figure 1.

**Table 1:** The top 15 research questions/topics

Final rank	Topic category	Research question/topic	Total points received
1	Chemotherapy	Identify molecular signatures to select patients who could be spared chemotherapy	643
2	Chemotherapy	Identify molecular features that indicate the optimal chemotherapy regimen (for example, combination or sequential, anthracyclin or not, and taxane or not)	450
3	DCIS	Determine the factors in DCIS and/or atypical ductal hyperplasia which lead to progression into invasive carcinoma	406
4	Stem cells	Determine the role of stem cells in breast cancer development, progression, and treatment sensitivity	404
5	Triple-negative/basal	Identify response/resistance mechanisms and thereby therapeutic targets for triple-negative breast cancer	369
6	Computing	Develop a system (computer and so on) that will integrate all the information gathered so far about breast cancer to build robust models for understanding the aetiopathogenesis, treatment, and prognosis of breast cancer	305
7	Prognosis	Identify which low-risk patients require no adjuvant therapy	301
8	New growth factor targets	Determine whether other growth factor pathways (such as epidermal growth factor receptor, insulin-like growth factor receptor, Notch, Hedeghog, Wnt, and other angiogenic pathways) are important targets for therapy	287
9	Genetics	Investigate which gene mutations in a tumour lead to metastases	236
10	Endocrine	Identify drugable targets that can be developed/exploited for therapeutic gain to overcome primary/secondary endocrine resistance	226
11	Consensus	Define consensus phenotyping procedures for specific molecular subtypes of breast cancer (immunohistochemistry, expression array, or reverse transcription-polymerase chain reaction signature genes)	201
12	Endocrine	Search for a more accurate and validated score of hormone sensitivity	180
13 (tie)	Imaging	Develop non-invasive techniques to diagnose and characterise primary breast tumours	171
13 (tie)	Endocrine	Determine whether there is a molecular profile (including PgR and HER2) that can distinguish patients likely to respond to tamoxifen versus an aromatase inhibitor	171
15	Herceptin: duration	Identify markers of the optimal duration of trastuzumab therapy	165

DCIS, ductal carcinoma *in situ*.

**Figure 2:**
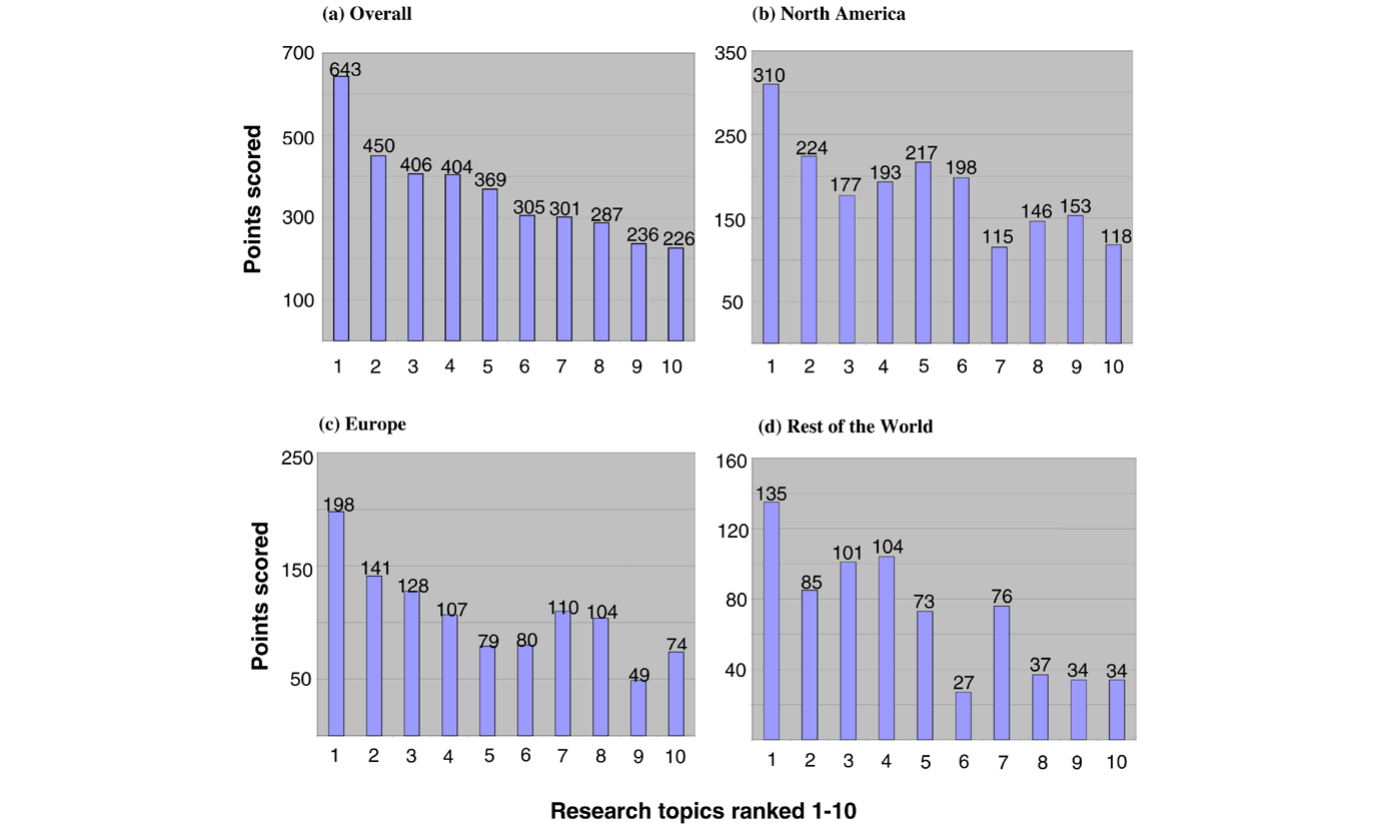
Distribution of votes for the top 10 topics by region of the world: **(a) **overall, **(b) **North America, **(c) **Europe, and **(d) **the rest of the world. The identity of each topic can be found by referring to the identical numbering in Table 1.

**Figure 3:**
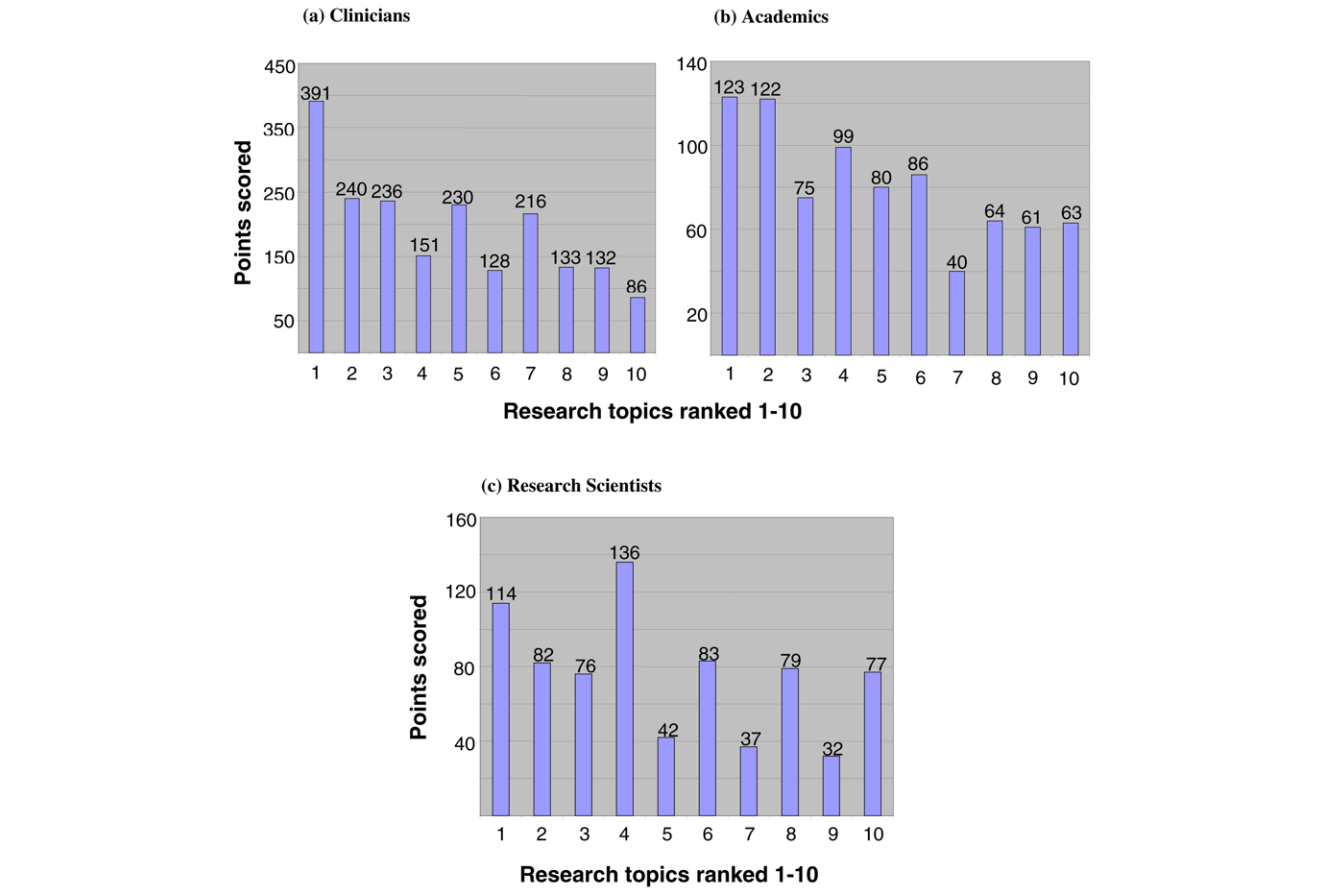
Distribution of votes for the top 10 topics by professional discipline: **(a) **clinicians, **(b) **academics, and **(c) **research scientists. The identity of each topic can be found by referring to the identical numbering in Table 1.

Table 1 shows that the top topic overall – with about 50% more voting points than any other – was to identify molecular signatures to select patients who could be spared chemotherapy. This was also the top question for each of the three regions of North America, Europe, and RoW (Figure 2). Clinicians rated this as clearly the top issue (Figure 3), but academics rated the overall second topic (to identify molecular features that indicate the optimal chemotherapy regimen) as only 1 point behind the No. 1 choice (123 points versus 122). Research scientists rated the overall No. 4 choice (to determine the role of stem cells in breast cancer development, progression, and treatment sensitivity) as the top question, with 136 votes versus 114 votes for the overall top question on the need for chemotherapy. This stem cell question was sixth in order of priority for the clinicians, third for the academics, and fourth overall.

Figures 2 and 3, respectively, reveal that geographic region of voting had only a modest effect on the voting pattern but that the professional discipline of the voter appeared to have a more marked influence, with substantial variation between the clinicians and the research scientists and, to a lesser degree, between the clinicians and the academics. An insufficient number of pathologists participated to assess these as a meaningful subgroup. At the bottom of the list of 70 priorities were two prognostic questions, one on the importance of young age and the other on the difference between lymphatic invasion and nodal status as prognostic factors.

## Discussion

In recent years, the interest in applying the results of laboratory studies to advance the treatment of breast cancer has increased substantially in so-called translational research. While this is a welcome development, there has been very wide targeting of resources. This may be based on many reasons, including the local opportunities available and a consideration of the most relevant/important questions to an individual's research. The rationale of the present study was that a wide consultation to identify priority questions in translational breast cancer research might help investigators, funding agencies, and journal reviewers/editors to prioritise their resources to address the most pressing clinical questions. The documentation of the questions widely considered to be of high priority may lead investigators to review the priority of their own research in part because such research may be seen by funding agencies as more worthy of support and high-impact journals may be more likely to accept it for publication.

In discussions before and during the project, the majority of consulted specialists felt the idea very worthwhile but a minority had strong reservations on a number of fronts. These included the possibility of voting being influenced by what is fashionable but of little real importance and also the possibility that, if this process did influence the research agenda, it might lead to the suppression of novel, but potentially highly important, ideas that inevitably would not feature in most voters' thoughts. The former of these issues probably can be addressed only by the passage of time, but the latter may be avoided by recognition of the possibility and an open-mindedness to new ideas/concepts as well as maintaining a focus on present priorities.

The use of the San Antonio and St Gallen e-mail databases as the primary contact sources aimed to achieve worldwide interest and voting, given the predominance of North American participants in San Antonio and of participants from other parts of the world (particularly Europe) in St Gallen. The results of the request for priority questions supported our starting position that there is a very wide opinion of what is important, given that more than 130 individual questions were logged and that these could be reduced by the steering committee to only 70 unique consolidated topics. It is important to consider whether 420 registrant voters is sufficient to provide a representative guide to worldwide opinion: this is supported by the similar voting patterns from the three geographical regions, with the top question being the same for all three and only minor variation existing between them for the other top issues.

Professional group appeared to influence voting more substantially. The stem cell question (No. 4 overall) gained only 151 points from clinicians compared with 391 for the topic of identifying patients who could avoid chemotherapy. In contrast, the respective points from research scientists were 136 and 114. These votes seem to be weighted according to the degree of interest in the topic for the respective professional group, the chemotherapy question being one that challenges clinicians on a frequent basis but that attracts less attention from scientists. The stem cell issue might be seen, particularly by scientists, as of central future importance to the progress in clinical breast cancer research. While this topic was not rated as unimportant by clinicians, the lower likelihood of its delivering clinically influential data in the near future might lead to its being considered less important.

It could be argued that if translational research is to be targeted by processes such as this consultation, the opinions of clinicians should be given greater weighting since it is they who will be directly challenged by the issues on a regular basis; the views of other specialists would be likely to be influenced by other factors unrelated to clinical importance. An examination of Figure 3 shows that such weighting was not necessary in this process since the clinician votes were rather similar to the overall voting pattern. It also seems likely that even without specifically creating a weighting, the use of two large e-mail listings from largely clinically orientated international meetings for making primary contacts will have resulted in a much larger proportion of clinician responses than if more general listings had been used.

The goal of identifying molecular signatures to select patients who could be spared chemotherapy being the top priority is likely to reflect the widespread recognition of the very good prognosis of patients diagnosed with breast cancer in recent years. This has resulted from earlier diagnosis largely as a result of screening and the major impact of the widespread application of hormone therapies to patients with oestrogen receptor-positive disease [4]. Thus, there is a large proportion of patients for whom any benefit in outcome as a result of adjuvant chemotherapy is likely to be so small as to be outweighed by concomitant toxic side effects. The voting for this topic supports the efforts of the TAILORx (Trial Assigning Individualized Options for Treatment [Rx]) and MINDACT (Microarray In Node-negative Disease may Avoid ChemoTherapy) investigators, who are investigating the clinical significance of the application of the OncotypeDX and MammoPrint molecular signatures, respectively [5,6].

In contrast, there are fewer well-publicised studies aimed at addressing the second most important topic, namely of identifying molecular features to select the optimal chemotherapy regimen. One of the issues here is that, while molecular signatures to address prognosis can be derived using samples from non-randomised sources, this second topic requires the assessment of large sample sets from clinical trials randomising different chemotherapy regimens. Collections of such tissues have only recently become the norm, so it is only in the last few years that we have achieved the ability to address what in reality is a series of questions on the comparison of multiple different regimens, one against the other. It should also be recognised that unless there is a very large difference between treatment effectiveness according to molecular subtype, a significant statistical interaction with treatment outcome is unlikely. The establishment of a good understanding of mechanisms of response and resistance to particular regimens in laboratory studies is likely to provide the greatest opportunity for providing such markers.

The third choice in the list of priorities was the goal of identifying factors in ductal carcinoma *in situ *and/or atypical ductal hyperplasia which lead to progression to invasive cancer. In common with the first priority, this can be viewed as attempting to identify patients who can avoid major therapy. The stem cell question (at No. 4) has been discussed above. The fifth priority – identifying the response and resistance mechanisms related to triple-negative/basal tumours – is one that itself has emanated from translational research in that the first expression array studies of breast cancer samples led to the identification of this group as an entity with poor prognosis and limited response to conventional therapies [1,7]. This is a clear example of the two-way nature of translational research, from the clinic to the laboratory and back to the clinic.

To address each of these topics in an instructive fashion, it is essential that there be close adherence to principles of good practise for translational research: (a) a clear understanding and declaration of the question to be addressed, (b) the selection of tissue sample sets suitable for directly addressing the question, (c) the application of well-validated reagents and methodologies to tissue samples characterised as being of high quality, (d) the linkage of the samples to accurate clinical information in cases in which the topic relates to clinical outcome (which it does in almost all of the top-priority cases), and (e) the testing of sufficiently numerous samples to meet prospectively derived statistical power calculations. If any of the above guidelines are not met, the study should be declared as hypothesis-generating. If the study does not select markers based on declared hypotheses, the initial study should be recognised as a training set, which requires validation on at least one appropriate, independent test set. The conclusions from these studies should be recognised as being applicable only within the populations studied (for example, with respect to age, menopausal status, and tumour size) unless subjected to further study to establish that the results may be generalised. Finally, to find its place in clinical practise, the new test may require prospective evaluation of its impact on clinical outcome.

The success and importance of the present exercise are difficult to judge but may be measured in part by citations of this article or the presentations of the results at the St Gallen meeting. This will determine whether it is repeated in the coming years, as there is no doubt that priorities will change with time.

## Abbreviations

RoW = the rest of the world.

## Competing interests

The authors declare that they have no competing interests.

## Authors' contributions

MD conceived the study and created its structure. All authors acted as members of a steering committee for the study, approved each stage of the study, and jointly consolidated the topic list. All authors read and approved the final manuscript.

## Supplementary Material

Additional file 1 Table of the 70 questions/topics in the prioritised according to the total number of points. Click here for file
