# Supplementary material for: International Web-based consultation on priorities for translational breast cancer research
Source: Breast Cancer Res. 2007 Nov 22;9(6):R81. doi: 10.1186/bcr1798 (PMC2246180; doi:10.1186/bcr1798)
Supplement: Additional file 1 — Table of the 70 questions/topics in the prioritised according to the total number of points. [file bcr1798-S1.DOC]

**S1:** The full consolidated list of 70 research topics / questions, ranked in order of the total numbers of points scored, as determined by the votes received.

| **FINAL RANK** | **Topic** | **Research question / topic** | **Points received as 1st choice** | **Points received as 2nd choice** | **Points received as 3rd choice** | **Points received as 4th choice** | **Points received as 5th choice** | **Points received as 6th choice** | **Total points received** |
| --- | --- | --- | --- | --- | --- | --- | --- | --- | --- |
| **1** | Chemotherapy | Identification of molecular signatures to select patients who could be spared chemotherapy. | 50 | 35 | 23 | 14 | 12 | 10 | **643** |
| **2** | Chemotherapy | Identify molecular features which indicate the optimal chemotherapy regimen (eg combination or sequential, anthracyclin or not, taxane or not). | 28 | 19 | 25 | 12 | 22 | 7 | **450** |
| **3** | DCIS | Determine the factors in DCIS and/or ADH leading to progression into invasive carcinoma. | 25 | 19 | 10 | 23 | 20 | 12 | **406** |
| **4** | Stem cells | Determine the role of stem cells in breast cancer development, progression and treatment sensitivity. | 31 | 17 | 15 | 13 | 10 | 14 | **404** |
| **5** | Triple negative / basal | Identify response/resistance mechanisms and thereby therapeutic targets for triple negative breast cancer. | 15 | 19 | 16 | 17 | 22 | 25 | **369** |
| **6** | Computing | Develop a system (computer etc) that will integrate all the information so far gathered about breast cancer to build robust models for understanding the aetiopathogenesis, treatment and prognosis of breast cancer. | 30 | 12 | 8 | 3 | 6 | 12 | **305** |
| **7** | Prognosis | Identifying which low risk patients require NO adjuvant therapy. | 19 | 14 | 13 | 15 | 7 | 6 | **301** |
| **8** | New growth factor targets | Determine if other growth factor pathways are important targets for therapy such as EGFR, IGFR, Notch, Hedeghog, Wnt and other angiogenic pathways. | 9 | 15 | 20 | 16 | 11 | 8 | **287** |
| **9** | Genetics | Investigate which gene mutations in a cancer lead to metastases. | 11 | 12 | 10 | 14 | 11 | 6 | **236** |
| **10** | Endocrine | Identify drugable targets that can be developed/ exploited for therapeutic gain to overcome primary/ secondary endocrine resistance. | 13 | 10 | 12 | 11 | 5 | 7 | **226** |

**Appendix 3 (continued)**

| **FINAL RANK** | **Topic** | **Research question / topic** | **Points received as 1st choice** | **Points received as 2nd choice** | **Points received as 3rd choice** | **Points received as 4th choice** | **Points received as 5th choice** | **Points received as 6th choice** | **Total points received** |
| --- | --- | --- | --- | --- | --- | --- | --- | --- | --- |
| **11** | Consensus | Define CONSENSUS phenotyping procedures for specific molecular subtypes of breast cancer (IHC, expression array or RT-PCR signature genes). | 10 | 12 | 8 | 12 | 5 | 3 | **201** |
| **12** | Endocrine | Search for a more accurate and validated score of hormone-sensitivity. | 10 | 13 | 8 | 5 | 3 | 2 | **180** |
| **13** | Imaging | Develop non-invasive techniques to diagnose and characterise primary breast cancers. | 9 | 8 | 7 | 6 | 11 | 9 | **171** |
| **14** | Endocrine | Determine if there is a molecular profile (including PgR and HER2) that can distinguish patients likely to respond to tamoxifen vs an AI. | 5 | 13 | 9 | 5 | 6 | 13 | **171** |
| **15** | Herceptin: duration | Identify markers of the optimal duration of trastuzumab therapy. | 6 | 7 | 12 | 6 | 8 | 12 | **165** |
| **16** | Chemotherapy | Determine how to suppress resistance to chemotherapy. | 9 | 7 | 7 | 9 | 8 | 1 | **161** |
| **17** | Endocrine | Seek molecular signatures that help define optimal sequencing of endocrine therapies for the individual patient. | 5 | 7 | 8 | 13 | 8 | 9 | **161** |
| **18** | Aetiology | Identify which women with a family history but no BRCA1/BRCA2 or P53 mutation really run an increased risk (and which don't) and at what age. | 5 | 4 | 8 | 9 | 16 | 19 | **160** |
| **19** | HER-based therapies | Determine mechanism of resistance and thereby identify targets to overcome resistance to HER-based therapies. | 4 | 5 | 11 | 12 | 6 | 13 | **154** |
| **20** | Brain metastases | How to predict who will develop brain metastases and isolate an effective treatment other than radiotherapy. | 6 | 6 | 9 | 4 | 9 | 12 | **144** |

**Appendix 3 (continued)**

| **FINAL RANK** | **Topic** | **Research question / topic** | **Points received as 1st choice** | **Points received as 2nd choice** | **Points received as 3rd choice** | **Points received as 4th choice** | **Points received as 5th choice** | **Points received as 6th choice** | **Total points received** |
| --- | --- | --- | --- | --- | --- | --- | --- | --- | --- |
| **21** | Neoadjuvant | Establish whether and how clinical or biological response to preoperative systemic therapy can optimise systemic endocrine or cytotoxic therapy. | 5 | 8 | 10 | 7 | 6 | 1 | **144** |
| **22** | Avastin | Molecular markers to select patients for Avastin, and other anti-VEGF therapies in the pipeline. | 4 | 7 | 5 | 10 | 7 | 20 | **143** |
| **23** | Risk assessment | More accurately determine the risks (including mammographic density) for breast cancer. | 10 | 4 | 7 | 6 | 5 | 5 | **141** |
| **24** | Prognosis | Establish whether gene signatures are better prognostic markers than classic St Gallen criteria after longer follow up (5-10yrs) or only the first 5 years. | 4 | 5 | 10 | 9 | 8 | 8 | **140** |
| **25** | Micromets | Determine the significance of minimal residual disease (MRD) in blood and bone marrow. | 1 | 9 | 8 | 6 | 13 | 7 | **134** |
| **26** | Prognosis | Define a reliable assay for defining patients who will not develop recurrent disease. | 7 | 8 | 4 | 5 | 5 | 2 | **125** |
| **27** | Prevention | Identify short-term surrogate markers (eg radiological, molecular etc) to determine who really benefits from chemoprevention. | 7 | 4 | 7 | 7 | 3 | 4 | **121** |
| **28** | Neoadjuvant | Determine which patients should receive preoperative chemotherapy. | 3 | 10 | 4 | 3 | 9 | 3 | **114** |
| **29** | Neoadjuvant | Identify biomarkers for predicting pathological complete response to chemotherapy. | 6 | 7 | 3 | 4 | 5 | 6 | **111** |
| **30** | Prognosis | Replace current TNM staging with molecular assay staging, avoiding the need for pathologic axillary staging. | 3 | 9 | 3 | 5 | 4 | 11 | **109** |

Appendix 3 (continued)

| **FINAL RANK** | **Topic** | **Research question / topic** | **Points received as 1st choice** | **Points received as 2nd choice** | **Points received as 3rd choice** | **Points received as 4th choice** | **Points received as 5th choice** | **Points received as 6th choice** | **Total points received** |
| --- | --- | --- | --- | --- | --- | --- | --- | --- | --- |
| **31** | Stem cells | Determine the effect of neoadjuvant therapy on the nature of tumour stem cells in the remaining tumour in the breast, and target these cells for therapy. | 5 | 8 | 3 | 3 | 4 | 3 | **102** |
| **32** | Herceptin | Determine whether there are identifiable subgroups that show differential benefit from Herceptin/lapatinib. | 3 | 2 | 5 | 10 | 6 | 8 | **98** |
| **33** | Prognosis | Develop more affordable version of OncotypeDx assay and extend this to node positive disease. | 2 | 6 | 3 | 6 | 8 | 2 | **90** |
| **34** | Aetiology | Clarify the role of pregnancy in the risk of sporadic vs hereditary breast cancer. | 3 | 3 | 5 | 4 | 8 | 7 | **88** |
| **35** | Triple negative/basal | Better define the molecular determinants of basal-like tumours. | 3 | 3 | 4 | 4 | 6 | 10 | **83** |
| **36** | Preoperative | Use "window-of-opportunity" clinical studies (ie time between biopsy and clinical resection of DCIS or early stage disease) to obtain biomarker and imaging evidence for activity of novel agents. | 1 | 3 | 3 | 9 | 9 | 3 | **81** |
| **37** | New growth factor targets | Better explore the role of the activated PI3k/Akt pathway and/or PTEN deletion in breast tumuors resistant to Herceptin, endocrine therapy and/or chemotherapy. | 1 | 2 | 7 | 7 | 5 | 4 | **79** |
| **38** | Radiotherapy | Select patients for whom accelerated partial breast irradiation or intraoperative radiotherapy is equivalent to whole breast irradiation. | 2 | 1 | 8 | 4 | 6 | 4 | **77** |
| **39** | Pharmacogenomics | What are the pharmacogenomic features that predict response and side effects of antiestrogens and aromatase inhibitors. | 3 | 3 | 4 | 5 | 3 | 6 | **76** |
| **40** | Prognosis | Identify serum markers that may indicate development of a resistant phenotype to direct an earlier switch to other treatment options. | 2 | 6 | 2 | 4 | 5 | 4 | **76** |

**Appendix 3 (continued)**

| **FINAL RANK** | **Topic** | **Research question / topic** | **Points received as 1st choice** | **Points received as 2nd choice** | **Points received as 3rd choice** | **Points received as 4th choice** | **Points received as 5th choice** | **Points received as 6th choice** | **Total points received** |
| --- | --- | --- | --- | --- | --- | --- | --- | --- | --- |
| **41** | HRT | Identify a subset of breast cancer patients suitable for HRT (hormone replacement therapy) to treat significant oestrogen-deprivation symptoms/dysfunctions during follow-up. | 1 | 3 | 2 | 7 | 6 | 10 | **72** |
| **42** | Surgery | Identify who could be cured without surgical resection. | 6 | 1 | 3 | 3 | 2 | 5 | **71** |
| **43** | Pharmacogenomics | Exploratory analysis of pharmacogenomics of toxicity and response to treatment. | 1 | 3 | 5 | 7 | 3 | 3 | **71** |
| **44** | Endocrine | Does duration and timing of chemotherapy depend upon the degree of endocrine responsiveness? | 3 | 5 | 4 | 1 | 3 | 2 | **70** |
| **45** | Herceptin: Topo2 | Confirm or refute whether amplification of Topoisomerase 2 can provide a guide to whether HER2 positive primary disease may be treated with Herceptin plus a non-anthracyclin without detriment to efficacy. | 2 | 2 | 4 | 2 | 7 | 6 | **64** |
| **46** | Contralateral | Determine via molecular and/or genetic signatures which patients remain at increased risk of secondary, contralateral breast malignancies despite hormonal therapy. | 1 | 3 | 5 | 4 | 4 | 2 | **63** |
| **47** | Endocrine | Can we identify molecular markers after short term preop endocrine therapy to determine who needs added chemotherapy ? | 1 | 4 | 5 | 2 | 3 | 4 | **62** |
| **48** | Radiotherapy | Identify those patients who develop local recurrence in spite of postoperative radio therapy after breast conserving operation. | 2 | 4 | 4 | 3 | 1 | 2 | **61** |
| **49** | Surgery | Study the biological events triggered by the act of surgery that might activate latent metastases. | 1 | 1 | 3 | 6 | 7 | 6 | **61** |
| **50** | Stem cells | Assess circulating (blood) and disseminated (marrow) tumour cells as window to study stem cells and predict late relapses. | 1 | 4 | 6 | 1 | 2 | 3 | **60** |

**Appendix 3 (continued)**

| **FINAL RANK** | **Topic** | **Research question / topic** | **Points received as 1st choice** | **Points received as 2nd choice** | **Points received as 3rd choice** | **Points received as 4th choice** | **Points received as 5th choice** | **Points received as 6th choice** | **Total points received** |
| --- | --- | --- | --- | --- | --- | --- | --- | --- | --- |
| **51** | HER-based therapies | Understand role of gene copy number or quantitative expression of HER2 RNA or protein as predictor of response to HER2 directed therapies. | 3 | 3 | 0 | 6 | 3 | 1 | **58** |
| **52** | Radiotherapy | Determine which patients we can exclude from radiotherapy or from a boost in obtaining local control as primary treatment. | 1 | 3 | 4 | 3 | 1 | 9 | **57** |
| **53** | Aetiology | Evaluate biological significance of androgens in development and progression of carcinoma and premalignant lesions. | 3 | 2 | 1 | 3 | 4 | 7 | **56** |
| **54** | Endocrine | Determine if tamoxifen is detrimental in women with hormone receptor positive HER2 overexpressing tumours. | 1 | 5 | 1 | 3 | 3 | 1 | **51** |
| **55** | Genetics | Whether to routinely scan large populations of breast cancer patients to increase the number of identified gene mutations relevant to familial breast cancer. | 3 | 1 | 3 | 3 | 0 | 6 | **50** |
| **56** | Ethnicity | Study the influence of race/ethnicity in outcomes. | 0 | 2 | 4 | 4 | 2 | 4 | **46** |
| **57** | Prevention | Learn why BRCA1 and BRCA2 mutations mainly increase risk for breast and ovarian, not other cancers (may be important insight for preventive measures). | 1 | 3 | 2 | 1 | 4 | 4 | **44** |
| **58** | Endocrine | Determine the role of ER beta in breast cancer. | 3 | 0 | 2 | 0 | 5 | 3 | **39** |
| **59** | Immunology | Determine the nature and characteristics of immune cell infiltrate in the specimens of breast tumours resected before and after chemotherapy. | 1 | 2 | 2 | 3 | 2 | 1 | **38** |
| **60** | Genetics | Determine the role of BRCA1 and 2 in non-hereditary breast cancer. | 3 | 0 | 1 | 2 | 3 | 3 | **37** |

**Appendix 3 (continued)**

| **FINAL RANK** | **Topic** | **Research question / topic** | **Points received as 1st choice** | **Points received as 2nd choice** | **Points received as 3rd choice** | **Points received as 4th choice** | **Points received as 5th choice** | **Points received as 6th choice** | **Total points received** |
| --- | --- | --- | --- | --- | --- | --- | --- | --- | --- |
| **61** | Prognosis | Developing nomograms predicting time of greatest risk of recurrence based on clinical and genetic factors. | 1 | 1 | 2 | 3 | 3 | 3 | **37** |
| **62** | Radiotherapy | Develop tests predictive of the risk of long-term complications from radiotherapy. | 0 | 2 | 2 | 2 | 2 | 2 | **30** |
| **63** | Endocrine | Determine the 5 & 10 yr DFS for adjuvant endocrine therapies can according to % cells expressing ER / PgR for node negative, N+ 1-3 and 4 or more and for each menopausal group separately. | 2 | 0 | 1 | 2 | 3 | 1 | **29** |
| **64** | Herceptin | Identify which patients are at most risk from cardiac toxicity from adjuvant trastuzumab. | 0 | 0 | 1 | 6 | 3 | 0 | **28** |
| **65** | Quality of life | Research which can prevent hair loss in young women suffering from breast cancer who are on chemotherapy. | 1 | 0 | 2 | 1 | 3 | 4 | **27** |
| **66** | Herceptin | Identify subgroup of patients who are essentially cured with addition of Herceptin to chemotherapy so that they can be spared from addition of other targeted agents such as Avastin. | 0 | 1 | 2 | 0 | 4 | 4 | **25** |
| **67** | Endocrine | Identify the degree of endocrine responsiveness in ER negative PgR positive tumors. | 0 | 0 | 2 | 3 | 2 | 2 | **23** |
| **68** | Risk assessment | Identify a target specific for pre-malignant breast lesions, the activation status of which could be measured by PET imaging to screen for women at risk of breast cancer. | 2 | 0 | 1 | 1 | 1 | 1 | **22** |
| **69** | Prognosis | Identify the difference between lymphatic invasion and nodal status as a prognostic factor. | 1 | 1 | 1 | 0 | 2 | 2 | **21** |
| **70** | Prognosis | Determine whether age under 35 is a genuine prognostic factor. | 0 | 2 | 1 | 1 | 1 | 1 | **20** |
